# The use of fluids and cardiovascular acting drugs in nordic intensive care units 2014 - an internet-based cross-sectional practice survey

**DOI:** 10.1186/2197-425X-3-S1-A155

**Published:** 2015-10-01

**Authors:** F Wickbom, M Kollind, E Wilkman, M Snäckestrand, A Oldner, A Perner, A Åneman, M Chew, Scandinavian Critical Care Trials Group

**Affiliations:** Hallands Sjukhus, Dept of Anaesthesia and Intensive Care, Halmstad, Sweden; Hallands Sjukhus, Dept of Surgery and Oncology, Halmstad, Sweden; Helsinki University Central Hospital, Dept of Anesthesiology, Intensive Care, Emergency Medicine and Pain Medicine, Helsinki, Finland; Sahlgrenska University Hospital, Dept. of Anaesthesia and Intensive Care, Gothenburg, Sweden; Karolinska University Hospital, Dept of Anaesthesiology, Surgical Services and Intensive Care, Stockholm, Sweden; Copenhagen University Hospital - Rigshospitalet, Dept of Intensive Care, Copenhagen, Denmark; Liverpool Hospital, Dept of Intensive Care, Sydney, Australia

## Introduction

Shock is common in the intensive care units (ICUs) and is treated with fluids and vasoactive drugs. In 2003 a Scandinavian practice survey was performed documenting an extensive use of colloids and dopamine (1). In the last decade new research has questioned these practices (2,3). Hemodynamic monitoring and evaluation is also under debate.

## Objectives

To identify current practice for the treatment of shock in Nordic ICUs. Primary outcomes were the type of vasopressor and/or inotropic (VP/I) drugs and resuscitation fluids used. Secondary outcomes was the type of hemodynamic monitoring used and desired target values.

## Methods

An observational descriptive cross-sectional study in intensive care units in Denmark, Finland, Norway and Sweden. Patients were included if in shock and older than 14 years with at least 4 hours of continuous VP/I -drug infusion during a 7-day inclusion period in July and August 2014. Pre-specified subgroup analysis for patients with sepsis and cardiac failure were performed.

## Results

171 patients were included. Median Sequential Organ Failure Assessment (SOFA) score was 8 and 80% were mechanically ventilated. 82% had received VP/I-drug therapy < 24h at inclusion. 84% received volume loading before onset of VP/I-drug treatment. Ringer´s solution was given in 90% of patients and starches in 3/143 patients. Hypotension was the most common indication for VP/I-drug therapy (95%). Noradrenaline was the most commonly used VP/I-drug, given in 98% of cases. 21% received treatment with more than one VP/I drug. Dopamine was used in 2/170 patients. 97% were monitored with invasive arterial blood pressure. MAP, followed by lactate and diuresis were considered the most important variables for monitoring. The pulmonary arterial catheter was used in 16%, arterial pulse wave analysis in 7% and echocardiography in 29%.

## Conclusions

Significant changes in fluid and vasopressor use have occurred within the last 10 years in Nordic ICUs. Ringer's solution and Noradrenaline are now used as first line treatment in early shock and the use of starches and dopamine is rare. Although most patients were monitored with invasive arterial blood pressure, more comprehensive hemodynamic monitoring was surprisingly used only in a minority of patients.

## Grant Acknowledgment

Supported by The Research Council of Halland County Council, Sweden.
